# Comparison of Microvascular Decompression and Two Isocenters Gamma Knife for the Treatment of Trigeminal Neuralgia Caused by Vertebrobasilar Dolichoectasia

**DOI:** 10.3389/fneur.2021.707985

**Published:** 2021-08-30

**Authors:** Zhen Zhao, SongShan Chai, JiaJing Wang, XiaoBing Jiang, ChuanSheng Nie, HongYang Zhao

**Affiliations:** ^1^Department of Neurosurgery, Union Hospital, Tongji Medical College, Huazhong University of Science and Technology, Wuhan, China; ^2^Department of Neurosurgery, Zhongnan Hospital of Wuhan University, Wuhan, China

**Keywords:** trigeminal neuralgia, vertebrobasilar dolichoectasia, microvascular decompression, gamma knife, pain relief

## Abstract

**Background:** Vertebrobasilar dolichoectasia (VBD) is one of the rare causes of trigeminal neuralgia (TN). The common surgical treatments for patients with TN caused by VBD (VBD-TN) are microvascular decompression (MVD) and Gamma Knife radiosurgery (GKRS). However, the therapeutic effects of the two methods have not been clinically compared, so this study was performed to evaluate the treatment outcomes of MVD and GKRS for patients with VBD-TN.

**Methods:** The retrospective study was performed from March 2011 to March 2019 in Wuhan Union Hospital. A total of 80 patients diagnosed with VBD-TN were included in this study, and they were divided into the MVD group (*n* = 46) and GKRS group (*n* = 34) according to the surgical methods. The imaging data, intraoperative findings, treatment outcomes, and complications of the two groups were analyzed and compared. Meanwhile, the influencing factors of the treatment effect are also explored on the two groups.

**Results:** Patients who underwent MVD were younger than patients who underwent GKRS (median ages were 61.1 and 65.4 years old, respectively, *p* = 0.03). The median follow-up was 61.1 months for the MVD group and 56.8 months for the GKRS group. The favorable outcomes [Barrow Neurological Institute (BNI) pain score, BNI scores I–II] occurred in 97.8% of patients treated with MVD and in 78.9% of patients treated with GKRS (*p* = 0.009). The favorable outcomes in the percentage of patients after MVD 1, 3, 5, and 7 years were 95.7, 85.1, 74.2, and 74.2%, respectively, whereas the corresponding percentages after GKRS were 76.5, 66.2, 56.6, and 47.2%, respectively (*p* = 0.031). The postoperative complications (except facial numbness) in the MVD group were higher than those in the GKRS group (*p* = 0.036), but the incidence of new and worsening facial numbness was lower in the GKRS group (*p* < 0.001).

**Conclusions:** MVD is superior to GKRS in obtaining and maintaining favorable outcomes for patients with VBD-TN, but it also comes with more complications other than facial numbness. Thus, the treatment program can be tailored to a patient's unique condition and wishes.

## Introduction

Trigeminal neuralgia (TN) is mainly characterized by recurrent paroxysmal electric shock-like or acupuncture-like pain in the distribution area of the trigeminal nerve that is composed of the eye branch (V1), the maxillary branch (V2), and the mandibular branch (V3), which usually lasts for a few seconds (1–3). The actions, including brushing teeth, gargling, washing face, chewing, and other activities, especially eating cold or hot food, can induce TN. The reason for TN has been widely considered that trigeminal nerve in the cerebellopontine angle area is compressed by the responsible blood vessels, resulting in demyelination of trigeminal nerve and short circuits of the impulse between the afferent and efferent nerves (4). Common offensive blood vessels include superior cerebellar artery (SCA), anterior inferior cerebellar artery (AICA), posterior inferior cerebellar artery (PICA), and mixed arterial veins. However, TN caused by the dolichoectasia change of the vertebrobasilar system merely accounts for 2–7% of all types of TN, which is relatively rare (5–8). The vertebrobasilar system is considered to be elongated if the basilar artery lies lateral to the margin of the clivus or dorsum sellae or if it bifurcates above the plane of the suprasellar cistern. Ectasia is regarded to be present when the diameter of the basilar artery is >4.5 mm (4).

Drug treatment should also be selected firstly once VBD-TN was diagnosed like TN caused by other blood vessels. The common drugs incorporated are carbamazepine, oxcarbazepine, phenytoin, and so on. When drugs cannot control the pain symptoms, surgical treatments should be considered as the alternative methods (9). There are various surgical techniques that have been used for these cases, including microvascular decompression (MVD), Gamma Knife radiosurgery (GKRS), percutaneous glycerol lesions, percutaneous balloon compression (PBC), and percutaneous radiofrequency thermocoagulation (PRT). MVD may be considered as the most definite and durable treatment method for VBD-TN (10). However, the postoperative outcomes of patients with VBD-TN, reported by some studies, are relatively unsatisfactory in terms of recurrence and complications (11). These unsatisfactory outcomes may be attributed to large-volume, stiff vessel walls, and high arterial flow, making it less mobile than small arteries (such as superior cerebellar artery reported as the most common offending artery) (12–14). Considering these, coupled with the extremely high risk of craniotomy, a lot of surgeons may prefer other surgical treatments; for instance, the GKRS is the most used alternative option, especially for elderly frail patients with organ dysfunction (2). However, single isocenter GKRS treatment for TN revealed poor pain relief, especially for trigeminal neuralgia caused by compression of big blood vessels (15). It has been reported that two isocenter GKRS can completely cover the offensive blood vessels and significantly improve the pain relief rate (16).

To the knowledge of previous studies, both MVD and two isocenter GKRS can effectively relieve the pain of patients for VBD-TN, but the effectiveness and safety of the two techniques that are worthy of research have not been reported. Therefore, we have retrospectively compared the treatment outcomes between MVD and GKRS for patients with VBD-TN.

## Patients and Methods

### Study Population

This study was conducted in accordance with the Declaration of Helsinki and approved by our institutional review board; the patients' informed consent was waived due to the retrospective nature of the study. Eighty consecutive patients with VBD-TN diagnosed between March 2011 and March 2019 were included in the study. They performed either MVD or two isocenter GKRS in Wuhan Union Hospital. In the same period, 960 patients diagnosed as classical TN with other offensive blood vessels including AICA, PICA, SCA, and petrosal vein were excluded. Moreover, patients diagnosed with secondary TN caused by tumor and inflammation in the cerebellopontine angle area were excluded, too. All patients were informed for the treatment programs of VBD-TN; the expected effects and complications of various treatment plans through interviews and the final choices of the surgical method were decided by the patients. All included patients were divided into the MVD group (*n* = 46) and GKRS group (*n* = 34) according to the surgical methods. The median age was 61.1 years in the MVD group and 65.4 in the GKRS group, ranging from 30 to 70 years old and 38 to 75 years old, respectively.

All patients were examined by using the 3.0-T magnetic resonance imaging (MRI) scanner (Magnetom Trio; Siemens AG, Erlangen, Germany) before surgery. MRI protocol included axial T1-weighted imaging, axial T2-weighted imaging, and axial 3D-SPACE sequence covering the posterior fossa. The sequence parameters on the 3D-SPACE sequence were as follows: repetition time (TR)/echo time (TE) = 1,000/150 ms, data matrix = 384 × 384, flip angle = 120°, slice thickness = 0.5 mm, interslice gap = 0 mm. The diagnostic criteria of VBD-TN are as follows: the maximum diameter of the vertebrobasilar system exceeds 4.5 mm, the basilar artery bifurcates above the level of the suprasellar cistern or near the side edge of the slope or the dorsal saddle, and the symptomatic side trigeminal nerve was compressed by the tortuous expansion of the vertebrobasilar system.

In addition, the clinical records of patients were reviewed, including clinical characteristics, pain laterality, pain distribution, neuroradiological presentations and their duration, intraoperative findings, treatment outcomes, and postoperative complications.

### MVD

The patients were positioned contralaterally after general anesthesia. The dura mater was cut after retrosigmoid craniectomy and then was suspended to release the appropriate amount of cerebrospinal fluid to provide space for operation. Through arachnoid dissection and slight elevation of the cerebellum, the neurovascular conflicts located at the trigeminal nerve root entry zone (REZ) were carefully observed using an operating microscope, and a 45° endoscope was used when necessary. It was found that all trigeminal nerve roots, with varying degrees, were compressed by the dolichoectasia of the vertebrobasilar system.

**Figure 1:**
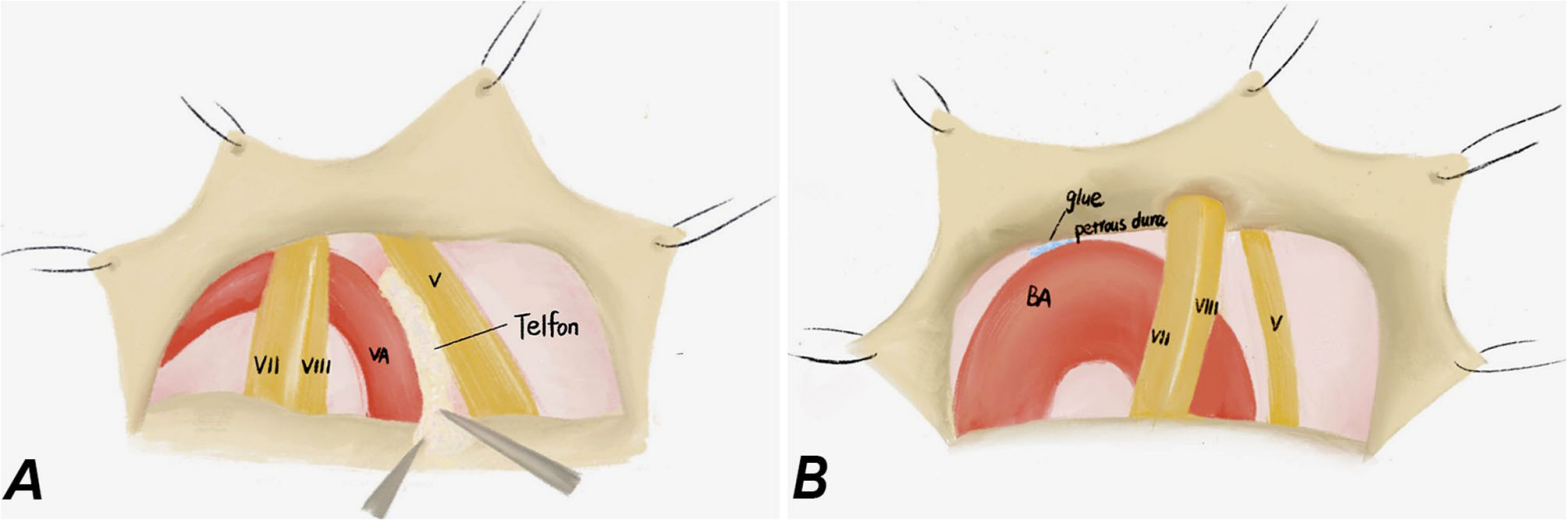
Intraoperative pictures of intervention method and transposition method. **(A)** Intervention method: the Teflon felt was inserted between the trigeminal nerve and VA. **(B)** Transposition method: the BA was fixed on the nearby petrous dura mater by using biological glue and a space was created between the trigeminal nerve and the BA.

In order to decompress the trigeminal nerve by dolichoectatic artery, two different MVD techniques were used in our study: interposition technique and transposition technique. In the interposition subgroup, we introduced the chopped Teflon felt implant into the conflicting neurovascular area between the artery and nervous structures, thereby separating the VBD from the trigeminal nerve (Figure 1A), while in the transposition subgroup, the proximal part of the vertebrobasilar artery was moved ventrally and cranially through the gap between the IX and VII–VIII nerves, and then fixed on the nearby petrous bone wall with biomedical glue (Figure 1B). It is worth noting that the perforating arteries should be protected to avoid secondary damage when suspended and to avoid twisting into angles for the responsible blood vessels (13, 17). All patients were firstly performed interposition technique, then according to the intraoperative situation, if the compression of the trigeminal nerve is insufficiently relieved or even worsened, the transposition technique should be performed.

### Radiosurgery Procedure

**Figure 2:**
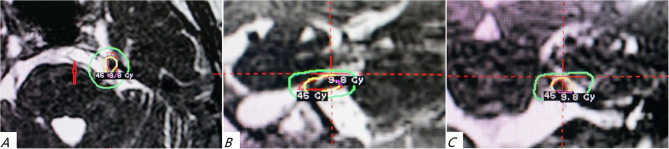
The two-isocenter gamma knife irradiation on the trigeminal nerve of patients with VBD-TN is shown in the axial **(A)**, sagittal **(B)**, and coronal **(C)** positions, respectively. The red arrow shows the tortuous and dilated vertebrobasilar artery. The figure clearly shows the full length of the cisternal trigeminal nerve on the affected side and shows the 50% isodose line (45 Gy, yellow circle) and the isodose line of 9.8 Gy (green circle).

The patients of the GKRS group were treated by using Leksell Model B Gamma Knife (Elekta Instruments, Stockholm, Sweden). The Leksell Model G stereotactic frame was installed on the skull after the patient received local anesthesia. Then, 3.0-T MRI was taken to clarify the relationship between the trigeminal nerve and its surrounding blood vessels, with 1 mm slice thickness, and no interval positioning scans were obtained. A 4-mm isocenter was placed adjacent to the trigeminal nerve root entry zone and another isocenter at the distal cisternal segment. Due to the closed proximity of the two targets, the 50% dose curves of the two isocenters often partially overlap and are connected to form a “cucurbit” shape. If the cisternal segment of the trigeminal nerve is severely moved by the vertebral artery or basilar artery and is difficult to be clearly displayed, one target is placed in the REZ area as much as possible according to the recognition situation, and the other target is often placed 2–3 mm behind the trigeminal nerve enters Meckel's cavity. The maximum central dose was 80–90 Gy, the isodose curve is 50%, and the dose of the pontine surface does not exceed 20 Gy (Figure 2).

### Evaluation of Treatment Outcome

Clinical follow-up information was obtained either by timed face-to-face assessment at our center or by regular telephone interviews. Barrow Neurological Institute pain intensity score (BNI) was used to assess patient pain (18). BNI score I, no pain, no medication; II, occasional pain, no medication; III, some pain, adequately controlled with medications; IV, some pain, not adequately controlled with medications; V, severe pain or no pain relief. Patients' pain intensity was assessed preoperatively, and at 1, 3, 6, and 12 months postoperatively and then yearly thereafter. We considered BNI scores I to II as representing favorable outcomes (pain-free or pain occasionally), and yet BNI scores III to V were defined as unfavorable outcomes. The BNI score of I was regarded as the pain-free outcome. Furthermore, we also sought patients if there are any complications such as facial numbness, dry eyes, hearing loss, and diplopia.

### Statistical Analysis

All statistical analyses were accomplished using SPSS software of version 25.0 (IBM Corp., Armonk, NY, USA). The continuous variables are reported as mean ± standard deviation (SD) or median and interquartile range (IQR), whereas categorical variables are presented as the absolute and relative frequencies. Statistical testing utilized non-parametric tests with the Mann–Whitney *U*-test and Kruskal–Wallis test for continuous variables, and the chi-square test or Fisher exact test for categorical variables. The interval of maintaining favorable outcomes that was defined as the time between no pain and detection of some pain controlled by drug (BNI III) or until the last follow-up was evaluated using the Kaplan–Meier method and compared by the log-rank test for the MVD group and GKRS group. Moreover, the correlation of treatment outcomes with clinical factors (age, sex, pain distribution, duration of symptoms, hypertension, median time to pain relief) was calculated using Pearson's chi-square test. The two-tailed *p*-values of < 0.05 were considered to indicate statistical significance.

## Results

### Patient Characteristics

The demographic and clinical characteristics of the patient population are shown in Table 1. There were 46 patients in the MVD group and 34 patients in the GKRS group included in this study, respectively. The average age of all participants was 63 years, and the proportion of male patients was 65%. The pain side is most common on the left side, accounting for 62.5% of cases. The branches of pain distribution are the most common in V2 + V3, accounting for about 37.5%. Demographic data showed that the age of patients performed by MVD was younger than the patients performed by GKRS, which based on the patient's choice (*p* = 0.03), whereas there was no significant difference in gender, pain side, basal BNI score, history of prior operation, hypertension, pain distribution, and duration of symptoms between the two groups of patients (all *p* > 0.05).

**Table 1:** Baseline characteristics of patients with VBD-TN in the MVD group and GKS group.

	**MVD (***n*** = 46)**	**GKRS (***n*** = 34)**	***P*** **-value**
Median age (years)	61.1	65.4	0.03
Gender (F/%)	18 (39.1%)	10 (29.4%)	0.37
Pain laterality (L/%)	30 (65.2%)	20 (58.8%)	0.75
With prior operation, *n* (%)	7 (15.2%)	8 (21.1%)	0.35
MVD	0	2	
GKRS	3	2	
PRT	3	4	
PBC	1	0	
Pain distribution			0.47
Single branch (V1 or V2 or V3)	16 (21.7%)	9 (26.5%)	
V1 + 2	3 (6.5%)	2 (5.9%)	
V2 + 3	20 (43.5%)	13 (38.2%)	
V1 + 2 + 3	7 (15.2%)	10 (29.4%)	
Duration of symptoms (years)	3.8 ± 2.3	4.6 ± 3.9	0.30
**Baseline BNI score**, ***n*****(%)**			
IV	24 (52.2%)	12 (35.3%)	0.17
V	22 (47.8%)	22 (64.7%)	
Offending VBA (radiological findings)			0.78
VA	37 (80.4%)	26 (76.5%)	
BA	9 (19.6%)	8 (23.5%)	
Arterial hypertension	17 (40.0%)	17 (50.0%)	0.25
Median follow-up (median)	61.1	56.8	0.66

*F, female; L, left; VBA, vertebrobasilar artery; VA, vertebral artery; BA, basilar artery; MVD, microvascular decompression; GKRS, Gamma Knife radiosurgery; PRT, percutaneous radiofrequency thermocoagulation; PBC, percutaneous balloon compression; BNI, Barrow Neurological Institute pain intensity score*.

### Pain Relief Outcomes

In terms of initial favorable outcomes (BNI scores I to II), all patients except one reached immediately initial favorable outcome after surgery in the MVD group. Among the 45 patients, 43 patients had BNI score I, and 2 patients had BNI score II. The remaining one patient had BNI score III. While there were only 27 patients who reached initial favorable outcomes in the GKRS group, 24 patients of them had BNI I, and 3 patients of them had BNI II. The remaining patients had unfavorable outcomes: three of them had BNI score III, one of them had BNI score IV, and three of them had BNI score V. It is obvious that the MVD group (97.8%) is statistically superior to the GKRS group (78.9%) on the initial favorable outcomes (*p* = 0.005, Table 2). Moreover, the time required to obtain initial favorable outcomes in the GKRS group was significantly longer than that in the MVD group (8 weeks vs. 0 day, *p* = 0.009, Table 2).

**Table 2:** Pain outcomes and complications of patients with VBD-TN after MVD and GKRS.

	**MVD (***n*** = 46)**	**GKRS (***n*** = 34)**	***p*** **-value**
Initial pain relief, *n* (%)			0.005
I	43 (93.5%)	24 (70.1%)	
II	2 (4.3%)	3 (8.8%)	
III	1 (2.2%)	3 (2.9%)	
IV	0	1 (40.0%)	
V	0	3 (8.8%)	
Initial favorable outcome (BNI I or II), *n* (%)	45 (97.8)	27 (79.4)	0.009
Median time to pain relief (IQR)	0 day (0–0 day)	8 weeks (1 week−6 months)	<0.001
New or worse numbness, *n* (%)	3 (6.5%)	16 (47.1%)	<0.001
Not bothersome	2 (4.3%)	12 (35.3)	
Somewhat bothersome	1 (2.2%)	2 (5.9)	
Very bothersome	0	2 (5.9)	
Other complications, *n* (%)			0.036
Newly development of dry eyes	1	2	–
Facial palsy	1	0	–
Taste hypoesthesia	2	0	–
Hearing loss	3	0	–
Diplopia	1	0	–
CSF leakage	1	0	–
Wound infection	2	0	–
BNI score at last follow-up, *n* (%)			0.011
I	36 (78.3%)	17 (50.0%)	
II	2 (4.3%)	3 (8.8%)	
III	2 (4.3%)	4 (11.8%)	
IV	2 (4.3%)	4 (11.8%)	
V	4 (8.7%)	6 (17.6%)	
Favorable outcome (BNI I or II) at last follow-up, *n* (%)	38 (82.6%)	20 (58.8%)	0.003

*MVD, microvascular decompression; GKRS, Gamma Knife radiosurgery*.

**Figure 3:**
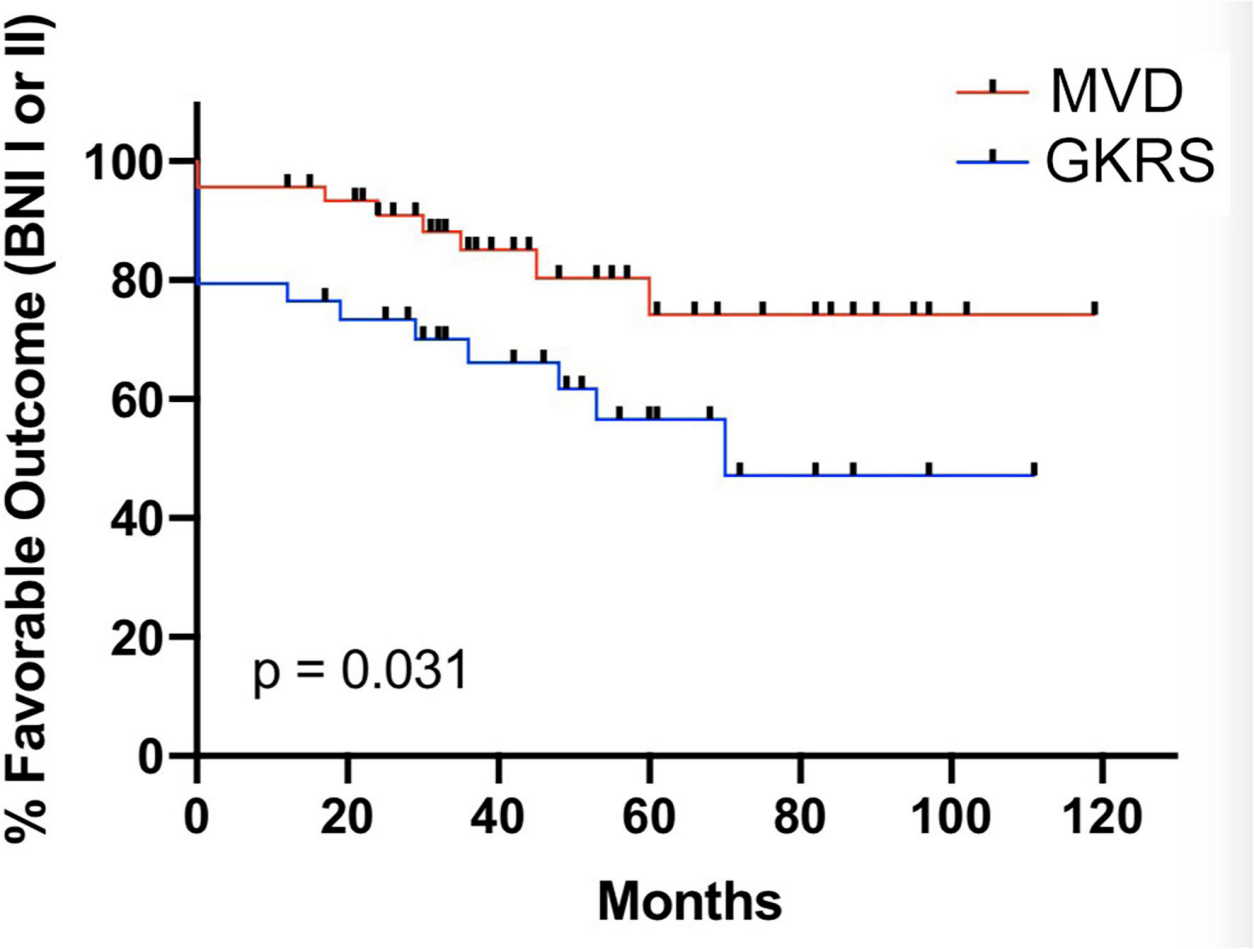
The Kaplan–Meier method was calculated the ability of maintaining the postoperative favorable outcome (BNI I–II) in the MVD group (red line) and GKRS (blue line) group (*p* = 0.031).

At the last follow-up, there were 38 patients (82.6%) in the MVD group and 20 patients (58.8%) in the GKRS group that maintained favorable outcomes. The MVD group showed to be significantly better compared with the GKRS group in terms of the ability to maintain favorable outcomes (*p* = 0.003, Table 2). In order to further investigate the long-term effect of the two surgical methods, Kaplan–Meier survival analysis was performed, which showed that the MVD group was significantly superior to the GKRS group for the time of maintaining favorable outcomes (log-rank test: *p* = 0.031, Figure 3). The favorable outcomes in the percentage of patients after MVD 1, 3, 5, and 7 years were 95.7, 85.1, 74.2, and 74.2%, respectively. The corresponding percentages after GKRS were 76.5, 66.2, 56.6, and 47.2%, respectively.

In the MVD group, the comparison of the influencing factors between patients with favorable outcomes (BNI I–II) and patients with unfavorable outcomes (BNI III–IV) found that there were no statistically significant differences in age, gender, pain laterality, surgical history, pain distribution, preoperative BNI scores, offensive blood vessels, and hypertension (all *p* > 0.05); only the MVD technique was significantly related to treatment outcomes (*p* = 0.037, Table 3). In the GKRS group, only new or worsening facial numbness after surgery was significantly different between favorable outcomes and unfavorable outcomes (*p* = 0.031, Table 4).

**Table 3:** Comparison of clinical factors between favorable outcomes and unfavorable outcomes in the MVD group.

	**Favorable outcome, BNI I or II**	**Unfavorable outcome, BNI III-V**	***P*** **-value**
No. of patients	38	8	
Mean age (years)	59.3	62.4	0.77
Gender (F/%)	14 (36.8%)	4 (50.0%)	0.69
Pain laterality (L/%)	24 (63.2%)	6 (75.0%)	0.45
With prior operation, *n* (%)	5 (13.2%)	2 (25.0%)	0.59
Pain distribution			0.93
Single branch (V1, V2 or V3)	13 (34.2%)	2 (25.0%)	
Multiple branch (V1 + 2; V2 + 3; V1 + 2 + 3)	25 (65.8%)	6 (75.0%)	
Duration of symptoms, (years)	3.6 ± 2.2	4.0 ± 2.5	0.44
**Baseline BNI score**, ***n*****(%)**			
IV	21 (55.3%)	3 (37.5%)	0.45
V	17 (44.7%)	5 (62.5%)	
Offending VBA (radiological findings)			0.95
VA	30 (78.9%)	7 (87.5%)	
BA	8 (21.1%)	1 (12.5%)	
Arterial hypertension	13 (34.2%)	4 (50.0%)	0.66
MVD method			0.037
Interposition technique	15 (39.5%)	7 (87.5%)	
Transposition technique	23 (60.5%)	1 (12.5%)	
New or worse numbness post-MVD, *n* (%)	2 (5.3%)	1 (12.5)	1

*F, female; L, center; VBA, vertebrobasilar artery; VA, vertebral artery; BA, basilar artery; MVD, microvascular decompression; BNI, Barrow Neurological Institute pain intensity score*.

**Table 4:** Comparison of clinical factors between favorable outcomes and unfavorable outcomes in GKRS group.

	**Favorable outcome, BNI I or II**	**Unfavorable outcome, BNI III-V**	***P*** **-value**
No. of patients	20	14	
Mean age (years)	66.1	64.2	0.77
Gender (F/%)	4 (20.0%)	6 (42.9%)	0.29
Pain laterality (L/%)	13 (65.0%)	7 (50.0%)	0.49
With prior operation, *n* (%)	4 (25.0%)	4 (28.6%)	0.69
Pain distribution			0.70
Single branch (V1, V2 or V3)	6 (30.0%)	3 (21.4%)	
Multiple branch (V1 + 2; V2 + 3; V1 + 2 + 3)	14 (70.0%)	11 (78.6%)	
Duration of symptoms, (years)	4.1 ± 3.4	4.8 ± 3.9	0.43
Baseline BNI score, *n* (%)			0.49
IV	6 (30.0%)	6 (42.9%)	
V	14 (70.0%)	8 (57.1%)	
Offending VBA (radiological findings)			1.00
VA	15 (75.0%)	11 (78.6%)	
BA	5 (25.0%)	3 (21.4%)	
Arterial hypertension	8 (40.0%)	9 (64.3%)	0.73
GKRS dose			0.72
80 Gy	0	1	
84 Gy	2	0	
86 Gy	3	1	
88 Gy	14	12	
90 Gy	1	0	
Median time to pain relief (IQR)	7 weeks (1 week−6 months)	10 weeks (0 day−7 months)	0.69
New or worse numbness post-GKRS, *n* (%)	13 (65.0%)	3 (21.4%)	0.031

*F, female; L, center; VBA, vertebrobasilar artery; VA, vertebral artery; BA, basilar artery; GKRS, Gamma Knife radiosurgery; BNI, Barrow Neurological Institute pain intensity score*.

### Postoperative Numbness

Table 2 demonstrates patients who received MVD significantly higher than patients who received GKRS in terms of new or worse numbness (three patients in the MVD group vs. sixteen patients in the GKRS group, *p* < 0.001). Among the three patients in the MVD group, two patients thought the numbness was not bothersome, and another suffered a little bothersome numbness, but no patients complained the numbness was very bothersome. In the GKRS group, likewise, most of them suffered numbness without being bothersome (eleven patients, 32.4%) and with being a little bothersome (two patients, 5.9%); only two patients complained the numbness was very bothersome.

### Other Complications

The comparison of other complications between the MVD and GKRS groups is shown in Table 2, which revealed that the MVD group was obviously higher than the GKRS group (eleven patients in the MVD group vs. two patients in the GKRS group, *p* = 0.036). These included three patients who experienced hearing loss with the median time of 2 years, taste hypoesthesia and wound infection were each complained by two patients, and new development of dry eyes, facial palsy, diplopia, and cerebrospinal fluid leakage were all experienced by one patient in the MVD group. Among them, a patient with dry eye did not require special intervention; a patient with diplopia was completely resolved spontaneously within 2 months without special treatment; patients with facial nerve palsy were treated with mecobalamin tablets (0.5 g, three times/day for 1 month), after which palsy symptom subsided completely; and patients with cerebrospinal fluid leakage were healed after surgical repairing. Of the two cases with hypogeusia, one case subsided spontaneously, and the other persisted for a long time. Hearing loss in all three patients occurred on the side of TN and received a comprehensive drug treatment including nimodipine, methylprednisolone, and neurotrophic agents. Besides, two of them also received adjuvant hyperbaric oxygen therapy. Their symptoms improved to varying degrees: one patient returned to normal hearing, while the other two cases developed permanent mild hearing impairment. However, in the GKRS group, only two patients complained about new development of dry eyes, and none of them required special intervention.

## Discussion

VBD, a rare arterial disease caused by vertebrobasilar tortuosity and expansion, can lead to obvious compression for the trigeminal nerve, which brings about the occurrence of TN. Some studies have reported that VBD-TN is more common in elderly male patients, usually on the left side, and tends to suffer from arterial hypertension (13, 19–22). In our study, the clinical characteristics in the MVD and GKRS groups were consistent with those findings. These characteristics can be explained by the general association of these three factors with atherosclerosis (23). It seems that long-term high blood flow has an impact on hemodynamics through atherosclerosis, which can cause arteries to bend and be tortuous (6). The vasodilatation may be related to the structural defects of the arterial wall in the inner elastic layer (24). The left side was the dominant pain side, which can be attributed to the asymmetry of the origin of the vertebrobasilar artery and hemodynamic factors (13, 25). The other findings are that the common pain distribution is a single branch and that V2 + V3 branches agree with those reported by Maarbjerg et al. (26). Those could be related to the position distribution of sensory fibers in the trigeminal nerve root.

The studies on the VBD-TN are still based on case reports and case sequences of a single regimen due to its relative rarity, and there is a lack of research on larger samples and comparison of treatment methods. Therefore, there is no standard methods to deal with the VBD-TN. Drug treatment is the preferred treatment, but when the pain is out of control, other forms of treatment are needed, such as MVD (5, 27), peripheral nerve resection (23), peripheral nerve alcohol injection (28), percutaneous glycerol lesions (29, 30), PBC (31, 32), GKRS (3, 33), and PRT (34, 35). MVD and GKRS are the more common treatment methods used in clinical practice nowadays due to good pain-relief outcomes acquired. MVD surgery has variety of forms; in general, there are two different modes for separating the offending arteries, including the interposition method and the transposition method (27). Therefore, MVD can fully push the vertebrobasilar artery away from the nerve by inserting a Teflon felt implant into the conflicting neurovascular area or fixing the blood vessels during the operation, thereby eliminating the neurovascular conflicts. Between the interposition technique and transposition technique, our team also made a comparison about them (27). The results found that patients with VBD-TN had a higher probability of maintaining a long-term pain-free status after MVD with the transposition technique compared to the interposition technique. Moreover, we noticed a trend that the incidence of new or worsening facial hypoesthesia in the interposition group was higher than the transposition technique in spite of that incidence of postoperative complications being similar between the groups. These results can be explained theoretically as follows: the interposition technique may not be able to obtain sufficient initial decompression effect by using only Teflon felt due to the hardness and size of the vertebrobasilar artery system, but a large amount of shredded Teflon felt and granuloma formation around the decompression site may put extra pressure on the TN. Fortunately, the offending arteries were fixed on the nearby petrous bone wall using biomedical glue with the transposition technique, which can avoid the formation of extra pressure, adhesions, and granulomas around the decompression site. Certainly, it is worth noting that excessive biomedical glue may cause overflow or dripping, which can lead to chemical vasculitis and peripheral nerve damage.

GKRS is a minimally invasive radiotherapy for the treatment of VBD-TN. According to the number of targets in the radiology, it can be divided into single isocenter (36), two isocenters (37), and multiple isocenters (3). The benefits of GKRS for TN have been recognized; furthermore, many patients with recurrent pain can relieve pain effectively after receiving the second GKRS treatment (2, 38, 39). This phenomenon led us to speculate that for some TN patients, especially obviously in patients with VBD-TN due to their wider neurovascular contact area, the volume of radiation that single-isocenter GKRS can provide may not be enough. Therefore, for better evaluating the effect of pain control and the complications, our center routinely performed the two-isocenter GKRS treatment for VBD-TN. Although the incidence of postoperative facial numbness is higher, only a small part of them are very annoying numbness (33).

Due to differences in technology, research methods, and patient selection among different institutions, the reported curative effect of surgical treatment for VBD-TN varies greatly. We, in this study, introduced the long-term experience of the surgical outcome based on a single institution using MVD and GKRS for VBD-TN and compared to their curative effect. To the knowledge of the authors, this study was also the first report comparing two different treatments of VBD-TN. We found that initial and long-term favorable outcomes were higher in patients treated with MVD. In addition, we also found that patients treated with GKRS have a higher age compared with patients treated with MVD, which means that they are more likely to have underlying diseases and physical weakness. Although age indicators are not specifically used to determine whether to perform the MVD method, they do reflect that patients and doctors jointly carry out potential preference selection to achieve excellent pain-control results and a reasonable incidence of adverse events.

The incidence of favorable outcomes in the MVD group was great (97.8%), and the rate of good pain control at 7 years after surgery was 74.2% in this study, which was similar to previously reported results. Furthermore, the transposition technique, consistent with our previous report (27), is a positive predictor of postoperative pain control. In the GKRS group, the incidence of early favorable outcomes was 78.9%, and the rate of good pain control at 7 years after surgery was 47.2%, which is consistent with the reported results by Chang et al. (3) but better than some other studies. All four cases treated with GKRS, reported by Lorenzoni et al. (40), had failed outcomes. Park et al. (2) reported that the 1-, 2-, and 5-year pain-relief rates for GKRS in VBD-TN patients remained at 53, 38, and 10%, respectively. The difference in treatment outcomes may be due to the two-isocenter and multiple-isocenter irradiation used in our study and that of Chang, respectively. Increasing the number of target points can cover a larger area of the affected myelinated nerve roots to increase the success rate of treatment, which is parallel to the GKRS treatment for TN secondary to brain tumors. Most studies have shown that postoperative facial numbness is a positive predictor of pain relief (41, 42), which was confirmed by our studies. In addition, age and duration of symptoms were not statistically different between good pain control and poor pain control in the two groups, although some studies have shown that older age (43, 44) and shorter duration of symptoms were predictors of postoperative pain relief (45, 46).

In terms of the time to obtain favorable outcomes, the MVD group was significantly more rapid and better than the GKRS group, for which the principles of the two technologies for relieving neurovascular conflicts completely differ. MVD surgery maintains a space between the trigeminal nerve and the responsible blood vessel, which can effectively interrupt the transmission of pulse force and free the trigeminal nerve from any contact, thus eliminating the abnormal discharge caused by the formation of a short circuit and stop the onset of pain (47, 48). The principle of two-isocenter GKRS is not only to conform to the trigeminal nerve root but also to increase the irradiated volume of the trigeminal nerve, so that the axons are fully degenerated and decomposed. This process takes a certain amount of time to achieve, so it does not have the immediate effect like MVD. In addition, failure of positioning, insufficient radiation, and tight neurovascular conflicts will also affect the pain relief effect.

When it comes to complications, the MVD group has a variety of types compared to the GKRS group, but the overall complication incidence is significantly lower. The results were understood very well by us. MVD surgery is invasive, especially the intricate neurovascular structure in the cerebellopontine area, which can easily cause side damage to nerves and blood vessels during the operation. These complications were not found in the GKRS group, and the most common complication was facial numbness, which is related to the working principle of the gamma knife. GKRS destroyed the structural integrity of the trigeminal nerve through diffuse demyelination, increases the pain threshold, and slows down the conduction of electrographic signals to relieve pain, but sometimes it is inevitable to damage non-pain sensory fibers (49). The postoperative complications may affect the quality of life of the patient. Therefore, a skilled surgeon required to perform rigorous preoperative evaluation, accurate target positioning, and appropriate radiation dose is the key element to ensuring the treatment effects and reducing the postoperative complications.

This study certainly had some limitations. First, the present study was performed with a single-center retrospective design and the sample size was small; thus, some uncertain bias might exist (50). Second, the patients were divided into two groups non-randomly, and there was a selection bias for the choice of surgical method. Third, follow-up deviation can also occur in that not all follow-up information of patients is obtained through face-to-face assessment. Fourth, the degree of neurovascular contact was not evaluated for which assessing from preoperative images alone is so subjective. Fifth, there are differences in age between the MVD and GKRS cohorts in our study; although we found no statistical difference in age between patients with favorable outcomes and unfavorable outcomes in two cohorts, we still cannot rule out the possibility that the age difference may lead to the observed difference between the MVD group and the GKRS group.

The results of this study showed that MVD could provide superior treatment effects than two-isocenter GKRS for patients with VBD-TN in terms of initial favorable outcomes and long-term pain control. Safely speaking, the GKRS with fewer serious postoperative complications is superior to MVD despite the higher incidence of facial numbness after GKRS. Certainly, this is a retrospective study with inevitable limitations; a randomized controlled trial with a large sample size would be required in the future.

## Data Availability Statement

The raw data supporting the conclusions of this article will be made available by the authors, without undue reservation.

## Ethics Statement

The studies involving human participants were reviewed and approved by Wuhan Union Hospital. The ethics committee waived the requirement of written informed consent for participants.

## Author Contributions

HYZ, ZZ, and SSC: conception and design. ZZ and JJW: collection and assembly of the data. SSC, ZZ, and JJW: data analysis and interpretation. All Authors: manuscript writing. All authors contributed to the article and approved the submitted version.

## Conflict of Interest

The authors declare that the research was conducted in the absence of any commercial or financial relationships that could be construed as a potential conflict of interest.

## Publisher's Note

All claims expressed in this article are solely those of the authors and do not necessarily represent those of their affiliated organizations, or those of the publisher, the editors and the reviewers. Any product that may be evaluated in this article, or claim that may be made by its manufacturer, is not guaranteed or endorsed by the publisher.
